# Supplementary material for: Evolutionary patterns of range size, abundance and species richness in Amazonian angiosperm trees
Source: PeerJ. 2016 Sep 6;4:e2402. doi: 10.7717/peerj.2402 (PMC5018673; doi:10.7717/peerj.2402)
Supplement: Table S1 — Amazonian tree genera in the phylogeny generated in this study, along with the species to which each rbcL and matK accession belongs and the associated Genbank accession numbers. For novel sequences, we also give the collection number. [file peerj-04-2402-s001.docx]

**Table S1:** Amazonian tree genera in the phylogeny generated in this study, along with the species to which each rbcL and matK accession belongs and the associated Genbank accession numbers. For novel sequences, we give the collection number.

| **Genus** | **Species rbcL** | **Species matK** | **Genbank accession rbcL** | **Genbank accession matK** | **Collection number** |
| --- | --- | --- | --- | --- | --- |
| Abarema | jupunba | macradenia | JQ626162 | GQ981925 |  |
| Abuta | sp. | sp. | JQ626102 | JQ626504 |  |
| Abutilon | sp. | NA | AM235023 |  |  |
| Acacia | bonariensis | greggii | KX640832 | AY386854 | BOLLC181CO |
| Acalypha | platyphylla | californica | AY794942 | EF135499 |  |
| Acanthosyris | falcata | NA | DQ329172 |  |  |
| Achatocarpus | nigricans | NA | KX640833 |  | BOLIN157 |
| Acosmium | dasycarpum | NA | U74255 |  |  |
| Adelia | virgata | ricinella | DQ997805 | AB268018 |  |
| Aegiphila | panamensis | NA | GQ981656 |  |  |
| Agonandra | silvatica | silvatica | JQ625908 | JQ626377 |  |
| Aiouea | longipetiolata | dubia | JQ625982 | EU153823 |  |
| Aiphanes | aculeata | aculeata | AY044626 | AM114641 |  |
| Albizia | julibrissin | julibrissin | Z70147 | AY386855 |  |
| Alchornea | sp. | trewioides | AY794956 | GU441801 |  |
| Alchorneopsis | floribunda | NA | AY794962 |  |  |
| Aldina | latifolia | NA | U74252 |  |  |
| Alexa | wachenheimii | wachenheimii | JQ625719 | JQ626338 |  |
| Alibertia | edulis | edulis | Z68843 | GQ981930 |  |
| Allamanda | cathartica | schottii | X91759 | DQ660495 |  |
| Allantoma | lineata | NA | AF077657 |  |  |
| Allophylus | angustatus | natalensis | JQ626023 | AY724268 |  |
| Alseis | lugonis | NA | Y18709 |  |  |
| Amaioua | guianensis | corymbosa | JQ625864 | GQ981931 |  |
| Amanoa | strobilacea | strobilacea | AY663562 | AY830258 |  |
| Ambelania | acida | acida | JQ626070 | JQ626477 |  |
| Ampelocera | ruizii | NA | KX640834 |  | BOLLC469 |
| Amphirrhox | longifolia | longifolia | JQ626095 | JQ626496 |  |
| Amphitecna | apiculata | NA | AF102640 |  |  |
| Anacardium | spruceanum | occidentale | JQ626226 | AY594459 |  |
| Anadenanthera | colubrina | peregrina | KX640835 | AF521814 | BOLIN013 |
| Anaxagorea | silvatica | silvatica | AY743439 | AY743477 |  |
| Andira | surinamensis | inermis | JQ625880 | GQ429072 |  |
| Aniba | rosaeodora | terminalis | KX640836 | JQ626487 | PER002 |
| Anisophyllea | disticha | fallax | AF127697 | AY935923 |  |
| Annona | prevostiae | prevostiae | JQ625732 | JQ626342 |  |
| Anthodiscus | pilosus | amazonicus | KX640837 | FJ670000 | PER004 |
| Antonia | ovata | NA | JQ625999 |  |  |
| Aparisthmium | cordatum | NA | AY794955 |  |  |
| Apeiba | glabra | glabra | JQ626190 | JQ626479 |  |
| Aphelandra | sinclairiana | sinclairiana | L01884 | GQ981937 |  |
| Aptandra | tubicina | tubicina | DQ790141 | DQ790178 |  |
| Apuleia | leiocarpa | leiocarpa | U74249 | EU361858 |  |
| Ardisia | crenata | crenata | L12599 | GU134982 |  |
| Aristolochia | arborea | brasiliensis | AB205586 | AB071819 |  |
| Aspidosperma | marcgravianum | marcgravianum | JQ626187 | FJ514763 |  |
| Astrocaryum | sciophilum | sciophilum | JQ626256 | JQ626555 |  |
| Astronium | ulei | graveolens | JQ625995 | AY594492 |  |
| Ateleia | herbert.smithii | arsenii | U74201 | GU220019 |  |
| Attalea | allenii | allenii | AJ404829 | AM114636 |  |
| Avicennia | marina | marina | AY008832 | AF477757 |  |
| Baccharis | halimifolia | neglecta | AF119188 | EU385326 |  |
| Bactris | humilis | gasipaes | AY044627 | AM114642 |  |
| Bagassa | guianensis | guianensis | JQ625997 | JQ626434 |  |
| Balizia | pedicellaris | NA | JQ625907 |  |  |
| Bambusa | sp. | beecheyana | M91626 | EU434244 |  |
| Banisteriopsis | hypericifolia | hypericifolia | AF344460 | AF344530 |  |
| Batesia | floribunda | floribunda | AY904375 | EU361869 |  |
| Bathysa | peruviana | NA | AM117206 |  |  |
| Batocarpus | amazonicus | NA | KX640838 |  | BOLLC433 |
| Bauhinia | guianensis | galpinii | JQ626034 | EU361875 |  |
| Beilschmiedia | brasiliensis | pendula | KX640839 | EU153824 | PER001 |
| Bellucia | pentamera | NA | AF215534 |  |  |
| Bertholletia | excelsa | NA | Z80178 |  |  |
| Besleria | affinis | NA | AF170226 |  |  |
| Bixa | orellana | NA | Y15139 |  |  |
| Blakea | schlimii | NA | EU711386 |  |  |
| Bocageopsis | mattogrossensis | multiflora | KX640840 | DQ018262 | PER007 |
| Bocoa | prouacensis | prouacensis | JQ626179 | JQ626415 |  |
| Boehmeria | biloba | NA | AJ390069 |  |  |
| Bombacopsis | nervosa | NA | JQ626327 |  |  |
| Bombax | buonopozense | NA | AF022118 |  |  |
| Bonnetia | roraimae | sessilis | AJ402930 | EF135509 |  |
| Bonyunia | minor | NA | AJ235818 |  |  |
| Borojoa | sp. | panamensis | AJ286694 | GQ981946 |  |
| Botryarrhena | venezuelensis | NA | KX640841 |  | PER008 |
| Bougainvillea | modesta | glabra | KX640842 | AY042560 | BOLIN088 |
| Bourreria | virgata | succulenta | AF258345 | DQ197229 |  |
| Brosimum | utile | rubescens | JQ626232 | JQ626346 |  |
| Brownea | ariza | coccinea | U74186 | EU361891 |  |
| Browneopsis | ucayalina | ucayalina | AM234233 | EU361894 |  |
| Brunellia | colombiana | sp. | AF291937 | AY935926 |  |
| Brunfelsia | americana | NA | AY206720 |  |  |
| Buchenavia | grandis | NA | JQ626298 |  |  |
| Buddleja | davidii | asiatica | AJ001757 | AJ429346 |  |
| Bunchosia | armeniaca | armeniaca | Z75274 | AF344533 |  |
| Burmeistera | domingensis | NA | DQ356148 |  |  |
| Bursera | inaguensis | fagaroides | L01890 | AY594462 |  |
| Buxus | sempervirens | sempervirens | DQ182333 | AF543728 |  |
| Byrsonima | crassifolia | crassifolia | AB233898 | AF344535 |  |
| Byttneria | aspera | NA | AY082352 |  |  |
| Cabralea | canjerana | NA | DQ238055 |  |  |
| Caesalpinia | pulcherrima | pulcherrima | U74190 | EU361906 |  |
| Calliandra | pittieri | eriophylla | AM234255 | EU025883 |  |
| Callicarpa | dichotoma | pentandra | L14393 | FM163264 |  |
| Calophyllum | sp. | antillanum | Z75672 | GU135089 |  |
| Calycophyllum | candidissimum | NA | X83627 |  |  |
| Calyptranthes | speciosa | pallens | JQ626314 | AF368201 |  |
| Capirona | decorticans | NA | JQ626324 |  |  |
| Capparis | maroniensis | amplissima | JQ625979 | EU371754 |  |
| Caraipa | tereticaulis | NA | KX640843 |  | PER011 |
| Carapa | procera | procera | JQ626164 | JQ626387 |  |
| Carica | papaya | papaya | M95671 | AY483221 |  |
| Cariniana | legalis | NA | Z80179 |  |  |
| Caryocar | glabrum | glabrum | JQ626035 | EF135515 |  |
| Caryodendron | orinocensis | orinocense | KX640844 | AB233753 | PER013 |
| Casearia | sylvestris | guianensis | JQ625967 | GQ981953 |  |
| Cassia | spruceana | grandis | JQ626269 | EU361909 |  |
| Cassipourea | guianensis | lanceolata | JQ625770 | FJ670038 |  |
| Castilla | ulei | NA | KX640845 |  | BOLET188 |
| Cathedra | acuminata | NA | JQ625808 |  |  |
| Catostemma | fragrans | fragrans | JQ626285 | JQ626568 |  |
| Cavanillesia | platanifolia | platanifolia | GQ981691 | GQ981956 |  |
| Cayaponia | americana | americana | DQ535737 | DQ536643 |  |
| Cecropia | obtusa | obtusifolia | JQ626251 | GQ429058 |  |
| Cedrela | odorata | odorata | AJ402938 | AY128182 |  |
| Cedrelinga | cateniformis | cateniformis | JQ625867 | AF521818 |  |
| Ceiba | samauma | pentandra | KX640846 | GQ981960 | BOLIN169 |
| Celtis | philippensis | philippensis | AY263941 | AY263925 |  |
| Centrolobium | microchaete | NA | KX640847 |  | BOLIN021 |
| Centropogon | granulosus | NA | EF174610 |  |  |
| Cespedesia | bonplandii | bonplandii | AJ420168 | EF135518 |  |
| Cestrum | nocturnum | elegans | AY206721 | AJ585891 |  |
| Chaetocarpus | schomburgkianus | castanocarpus | JQ626205 | AY552460 |  |
| Chamaecrista | fasciculata | fasciculata | U74187 | AY386955 |  |
| Chamaedorea | microspadix | fragrans | AJ404787 | DQ178674 |  |
| Chaunochiton | kappleri | kappleri | JQ626175 | DQ790179 |  |
| Cheiloclinium | cognatum | cognatum | JQ626275 | JQ626564 |  |
| Chelyocarpus | repens | ulei | AY012457 | AM114562 |  |
| Chimarrhis | turbinata | turbinata | JQ626106 | JQ626508 |  |
| Chiococca | alba | alba | L14394 | AY538378 |  |
| Chionanthus | virginicus | retusus | DQ673309 | EU409442 |  |
| Chione | sylvicola | NA | AM117215 |  |  |
| Chlorocardium | venenosum | NA | KX640848 |  | PER017 |
| Chomelia | sp. | NA | Y11846 |  |  |
| Chorisia | speciosa | NA | KX640849 |  | BOLIN043 |
| Chrysobalanus | icaco | icaco | L11178 | EF135519 |  |
| Chrysochlamys | membranacea | NA | AF518380 |  |  |
| Chrysophyllum | lucentifolium | argenteum | JQ626114 | JQ626548 |  |
| Cinchona | officinalis | officinalis | AY538480 | AY538381 |  |
| Cinnamomum | camphora | camphora | L12641 | AJ247154 |  |
| Cissus | quadrangularis | NA | AJ419720 |  |  |
| Citrus | paradisi | reticulata | AJ238407 | FJ716729 |  |
| Clarisia | racemosa | NA | KX640850 |  | PER018 |
| Clavija | eggersiana | NA | L12608 |  |  |
| Cleidion | castaneifolium | NA | AY794936 |  |  |
| Clerodendrum | fragrans | thomsonae | L11689 | AF315298 |  |
| Clethra | alnifolia | alnifolia | L12609 | AJ429281 |  |
| Clidemia | rubra | septuplinervia | AF215535 | GQ981968 |  |
| Clitoria | ternatea | NA | U74237 |  |  |
| Clusia | grandiflora | grandiflora | JQ626019 | FJ514669 |  |
| Cnidoscolus | urens | aconitifolius | AY794874 | AB268041 |  |
| Coccoloba | mollis | mollis | JQ626225 | JQ626541 |  |
| Cochlospermum | vitifolium | NA | AF022129 |  |  |
| Cojoba | rufescens | rufescens | GQ981709 | GQ981971 |  |
| Colubrina | reclinata | asiatica | AJ390065 | GU135023 |  |
| Combretum | goldieanum | apiculatum | FJ381801 | EU214221 |  |
| Commiphora | habessinica | NA | U39276 |  |  |
| Compsoneura | atopa | atopa | EU090508 | EU090469 |  |
| Conceveiba | guianensis | guianensis | JQ626138 | JQ626450 |  |
| Connarus | conchocarpus | NA | U06798 |  |  |
| Conostegia | bracteata | NA | GQ981710 |  |  |
| Cordia | sagotii | sagotii | JQ626197 | JQ626469 |  |
| Corythophora | rimosa | NA | AF077653 |  |  |
| Cosmibuena | grandiflora | grandiflora | AM117220 | AY538385 |  |
| Couepia | guianensis | habrantha | JQ626008 | FJ514651 |  |
| Couma | guianensis | guianensis | JQ626239 | DQ660512 |  |
| Couratari | guianensis | multiflora | JQ626273 | JQ626511 |  |
| Couroupita | guianensis | NA | Z80181 |  |  |
| Coussarea | macrophylla | curvigemmia | Y11847 | GQ981974 |  |
| Coutarea | hexandra | hexandra | AM117221 | GQ981975 |  |
| Crepidospermum | goudotianum | NA | JQ626087 |  |  |
| Crescentia | portoricensis | NA | AF102643 |  |  |
| Crotalaria | incana | pumila | Z70134 | AY386867 |  |
| Croton | setiger | gratissimus | AY794910 | EU214230 |  |
| Crudia | sp. | choussyana | KX640851 | EU361921 | PER021 |
| Cupania | scrobiculata | scrobiculata | JQ625966 | EU720671 |  |
| Cuphea | llavea | NA | AF495773 |  |  |
| Cyclolobium | blanchetianum | NA | KX640852 |  | BOLLC377 |
| Cymbopetalum | brasiliense | torulosum | AY841608 | AY743480 |  |
| Cynometra | iripa | mannii | AY289677 | EU361925 |  |
| Cyrilla | racemiflora | racemiflora | L01900 | AJ429282 |  |
| Cyrillopsis | paraensis | paraensis | JQ625942 | FJ670024 |  |
| Dacryodes | cuspidata | cuspidata | JQ626006 | JQ626441 |  |
| Dalbergia | hupeana | sissoo | U74236 | GU135125 |  |
| Dalechampia | spathulata | spathulata | AY788172 | EF135525 |  |
| Dendrobangia | boliviana | boliviana | JQ626064 | JQ626474 |  |
| Derris | laxiflora | reticulata | U74234 | AB504375 |  |
| Dialium | guianense | guianense | JQ625793 | EU361930 |  |
| Dialypetalanthus | fuscescens | NA | AF206761 |  |  |
| Dichapetalum | macrocarpum | macrocarpum | AF089764 | EF135527 |  |
| Diclinanona | tessmannii | NA | KX640853 |  | PER023 |
| Dicorynia | guianensis | guianensis | JQ626129 | EU361931 |  |
| Dictyocaryum | lamarckianum | lamarckianum | AY012479 | AM114616 |  |
| Dicymbe | uaiparaensis | altsonii | KX640854 | EU361932 | PER024 |
| Dioclea | virgata | NA | AF308709 |  |  |
| Diodia | sarmentosa | NA | AJ288600 |  |  |
| Dioscorea | wallichii | alata | AY939888 | AB040208 |  |
| Diospyros | carbonaria | carbonaria | JQ626229 | JQ626407 |  |
| Diploon | cuspidatum | cuspidatum | JQ626045 | JQ626461 |  |
| Diplotropis | purpurea | brasiliensis | JQ625878 | AY386939 |  |
| Dipteryx | odorata | oleifera | JQ625725 | GQ981983 |  |
| Discophora | guianensis | guianensis | JQ625904 | JQ626375 |  |
| Drymonia | stenophylla | NA | AF170232 |  |  |
| Drypetes | fanshawei | roxburghii | JQ625900 | EF135530 |  |
| Duckeodendron | cestroides | NA | AF206763 |  |  |
| Duguetia | surinamensis | surinamensis | JQ625927 | JQ626384 |  |
| Dulacia | guianensis | guianensis | JQ625816 | JQ626357 |  |
| Duroia | eriopila | eriopila | JQ626024 | JQ626449 |  |
| Dussia | discolor | macroprophyllata | JQ625757 | AY386903 |  |
| Dystovomita | brasiliensis | NA | AF518387 |  |  |
| Ecclinusa | ramiflora | NA | JQ626076 |  |  |
| Elaeis | oleifera | oleifera | AY012509 | EU016887 |  |
| Elaeoluma | nuda | nuda | JQ626242 | JQ626547 |  |
| Elvasia | calophyllea | NA | FJ670171 |  |  |
| Emmotum | fagifolium | fagifolium | JQ626244 | JQ626549 |  |
| Endlicheria | melinonii | melinonii | JQ625787 | JQ626354 |  |
| Enterolobium | schomburgkii | schomburgkii | JQ626149 | GQ981984 |  |
| Eperua | falcata | falcata | JQ626198 | JQ626458 |  |
| Ephedranthus | parviflorus | sp. | AY841615 | AY841396 |  |
| Eriotheca | longitubulosa | longitubulosa | JQ626270 | JQ626561 |  |
| Erisma | floribundum | floribundum | JQ626108 | JQ626510 |  |
| Erythrina | crista.galli | NA | Z70170 |  |  |
| Erythroxylum | spruceanum | panamense | KX640855 | GQ981987 | PER028 |
| Eschweilera | coriacea | coriacea | JQ626161 | JQ626454 |  |
| Eugenia | patrisii | feijoi | JQ626196 | JQ626380 |  |
| Euphorbia | polychroma | polychroma | L13185 | EF135539 |  |
| Euplassa | inaequilis | inaequalis | KX640856 | EU642689 | PER029 |
| Euterpe | oleracea | oleracea | JQ626233 | JQ626542 |  |
| Exellodendron | barbatum | NA | JQ625744 |  |  |
| Exostema | caribaeum | lineatum | AY205358 | AY538387 |  |
| Faramea | pedunculata | occidentalis | JQ626208 | GQ981990 |  |
| Ferdinandusa | paraensis | paraensis | JQ625906 | JQ626376 |  |
| Ficus | genima | sp. | KX640857 | JQ626578 | PER032 |
| Fusaea | longifolia | longifolia | JQ626101 | JQ626502 |  |
| Galipea | ciliata | NA | KX640858 |  | BOLIN001 |
| Garcinia | madruno | latissima | JQ626234 | FJ670008 |  |
| Gasteranthus | corallinus | NA | AF170233 |  |  |
| Gavarretia | terminalis | NA | AY794953 |  |  |
| Geissanthus | sp. | NA | AF213810 |  |  |
| Geissospermum | laeve | laeve | JQ625891 | DQ660517 |  |
| Genipa | americana | americana | Z68839 | GQ982000 |  |
| Geonoma | oxycarpa | congesta | AY044623 | AM114655 |  |
| Gloeospermum | longifolium | longifolium | AB354413 | AB354485 |  |
| Glycydendron | amazonicum | amazonicum | JQ626237 | JQ626544 |  |
| Gonzalagunia | affinis | NA | Y11848 |  |  |
| Gordonia | axillaris | NA | AF421092 |  |  |
| Gossypium | robinsonii | NA | L13186 |  |  |
| Gouania | mauritiana | NA | AJ390040 |  |  |
| Goupia | glabra | glabra | JQ626141 | EF135544 |  |
| Graffenrieda | rotundifolia | NA | AF215532 |  |  |
| Grias | cauliflora | NA | AF077652 |  |  |
| Guapira | standleyana | standleyana | GQ981748 | GQ982001 |  |
| Guarea | silvatica | silvatica | JQ626002 | JQ626438 |  |
| Guatteria | guianensis | guianensis | JQ626291 | JQ626573 |  |
| Guatteriopsis | hispida | kuhlmannii | DQ861837 | DQ861742 |  |
| Guazuma | ulmifolia | ulmifolia | GQ981753 | GQ982003 |  |
| Guettarda | acreana | speciosa | JQ626041 | AY538389 |  |
| Gurania | makoyana | makoyana | DQ535814 | DQ536680 |  |
| Gustavia | hexapetala | NA | JQ626207 |  |  |
| Gymnanthes | lucida | NA | AY794858 |  |  |
| Hamelia | papillosa | papillosa | AY538487 | AY538391 |  |
| Hampea | appendiculata | NA | GQ981758 |  |  |
| Haploclathra | cordata | NA | AY625017 |  |  |
| Hasseltia | floribunda | floribunda | GQ981759 | EF135546 |  |
| Hebepetalum | humiriifolium | NA | JQ625741 |  |  |
| Hedyosmum | sprucei | arborescens | AY236846 | DQ401339 |  |
| Heisteria | densifrons | densifrons | JQ626157 | JQ626520 |  |
| Helicostylis | elegans | tomentosa | KX640859 | JQ626514 | PER035 |
| Helicteres | angustifolia | NA | AY082356 |  |  |
| Heliocarpus | americanus | NA | KX640860 |  | BOLLC334 |
| Heliotropium | arborescens | aegyptiacum | L14399 | EU599646 |  |
| Hemidiodia | ocimifolia | NA | AJ288607 |  |  |
| Henriettea | martiusii | NA | EU711391 |  |  |
| Henriettella | flavescens | NA | JQ626220 |  |  |
| Hernandia | albiflora | ovigera | L77210 | AJ966799 |  |
| Herrania | purpurea | purpurea | GQ981762 | GQ982011 |  |
| Heteropterys | thyrsoidea | ciliata | AF344480 | AF344550 |  |
| Hevea | guianensis | brasiliensis | JQ626136 | AB268047 |  |
| Hibiscus | punaluuensis | syriacus | AJ233121 | AF345329 |  |
| Hieronyma | alchorneoides | alchorneoides | JQ626093 | FJ514743 |  |
| Hillia | triflora | NA | X83642 |  |  |
| Himatanthus | sp. | sp. | JQ625987 | JQ626428 |  |
| Hippotis | sp. | NA | Y11850 |  |  |
| Hiraea | smilacina | bahiensis | AF344484 | AF344552 |  |
| Hirtella | suffulta | suffulta | JQ625956 | JQ626404 |  |
| Homalium | racemosum | foetidum | AJ418822 | AB233830 |  |
| Hortia | excelsa | NA | JQ625842 |  |  |
| Huertea | glandulosa | NA | AY646109 |  |  |
| Humiria | balsamifera | balsamifera | AB233889 | AY935932 |  |
| Humiriastrum | subcrenatum | subcrenatum | JQ626167 | JQ626522 |  |
| Hura | crepitans | crepitans | KX640861 | FJ670012 | BOLLC337 |
| Hybanthus | prunifolius | prunifolius | GQ981766 | GQ982014 |  |
| Hydrolea | ovata | ovata | L14293 | AJ429356 |  |
| Hymenaea | courbaril | courbaril | JQ625969 | AY386906 |  |
| Hymenolobium | pulcherrium | mesoamericanum | KX640862 | AY386934 | PER038 |
| Hyperbaena | ilicifolia | NA | FJ026487 |  |  |
| Ilex | vomitoria | aquifolium | AF119184 | AF542607 |  |
| Inga | alba | paraensis | JQ625728 | JQ626408 |  |
| Ipomoea | wrightii | batatas | AY100957 | AJ429355 |  |
| Iriartea | deltoidea | deltoidea | KX640863 | AM114617 | PER040 |
| Iryanthera | sagotiana | sagotiana | JQ625975 | JQ626420 |  |
| Isertia | spiciformis | spiciformis | JQ625738 | JQ626345 |  |
| Ixora | biflora | NA | Z68866 |  |  |
| Jacaranda | copaia | copaia | JQ626146 | JQ626519 |  |
| Jacaratia | corumbensis | digitata | AF405245 | AY461574 |  |
| Jacquinia | keyensis | NA | AF213817 |  |  |
| Jatropha | capensis | integerrima | AM234978 | AB233775 |  |
| Joannesia | princeps | NA | AJ418808 |  |  |
| Joosia | aequatoria | umbellifera | AY538491 | AY538396 |  |
| Justicia | americana | NA | L14401 |  |  |
| Klarobelia | cauliflora | megalocarpa | AY841627 | AY518866 |  |
| Kutchubaea | semisericea | NA | AM117235 |  |  |
| Lacistema | grandifolium | grandifolium | JQ625828 | JQ626360 |  |
| Lacmellea | aculeata | aculeata | JQ626053 | JQ626466 |  |
| Lacunaria | jenmanii | NA | JQ626224 |  |  |
| Ladenbergia | macrocarpa | macrocarpa | AY538496 | AY538400 |  |
| Laetia | procera | procera | JQ625734 | JQ626344 |  |
| Lafoensia | acuminata | punicifolia | AY905411 | GQ982030 |  |
| Lantana | camara | camara | AF156736 | GU134977 |  |
| Leandra | mexicana | NA | AF215536 |  |  |
| Lecointea | peruviana | peruviana | AM234260 | EU361990 |  |
| Lecythis | persistens | persistens | JQ626036 | JQ626453 |  |
| Leonia | glycycarpa | glycycarpa | JQ626288 | JQ626572 |  |
| Licania | canescens | hypoleuca | JQ625929 | GQ982032 |  |
| Licaria | chrysophylla | chrysophylla | JQ625945 | FJ514617 |  |
| Lindackeria | spPennington | laurina | AJ418800 | GQ982034 |  |
| Lissocarpa | guianensis | guianensis | EU980794 | AJ429287 |  |
| Lonchocarpus | hermannii | heptaphyllus | AB045809 | GQ982035 |  |
| Loreya | arborescens | NA | JQ626318 |  |  |
| Lozania | pittieri | pittieri | GQ981790 | FJ670026 |  |
| Luehea | paniculata | seemannii | KX640864 | GQ982036 | BOLLC121 |
| Lueheopsis | rugosa | NA | JQ626279 |  |  |
| Lunania | parviflora | parviflora | KX640865 | AB233832 | PER043 |
| Lycianthes | sp. | rantonnei | FJ914173 | EF537320 |  |
| Mabea | piriri | piriri | JQ625733 | JQ626343 |  |
| Macairea | radula | NA | EU711394 |  |  |
| Machaerium | lunatum | NA | U74248 |  |  |
| Machaonia | sp. | NA | AM117244 |  |  |
| Maclura | pomifera | NA | D86318 |  |  |
| Macoubea | guianensis | guianensis | JQ625771 | JQ626352 |  |
| Macrocentrum | repens | NA | AF215513 |  |  |
| Macrocnemum | roseum | roseum | GQ981792 | GQ982037 |  |
| Macrolobium | acaciifolium | bifolium | U74191 | EU361996 |  |
| Magnolia | tripetala | albosericea | AF206791 | AF123464 |  |
| Mahurea | exstipulata | NA | AY625018 |  |  |
| Malanea | sp. | NA | AM117245 |  |  |
| Malmea | dielsiana | dielsiana | AY238955 | AY238964 |  |
| Malouetia | guianensis | guianensis | JQ625814 | JQ626356 |  |
| Malpighia | romeroana | romeroana | GQ981793 | GQ982038 |  |
| Manihot | esculenta | esculenta | AB233880 | AB233776 |  |
| Manilkara | bidentata | bidentata | JQ626263 | JQ626559 |  |
| Maprounea | guianensis | guianensis | JQ625792 | EF135563 |  |
| Maquira | guianensis | guianensis | JQ626247 | GQ982039 |  |
| Margaritaria | tetracocca | cyanosperma | Z75675 | AY552435 |  |
| Marila | racemosa | NA | AY625008 |  |  |
| Mascagnia | anisopetala | rivularis | AF344494 | AB233797 |  |
| Matayba | laevigata | laevigata | JQ625852 | EU720676 |  |
| Matelea | hirsuta | quirosi | AJ419747 | Z98196 |  |
| Matisia | ochrocalyx | NA | KX640866 |  | PER048 |
| Mauritia | flexuosa | flexuosa | KX640867 | AM114545 | PER050 |
| Mauritiella | sp. | NA | KX640868 |  | PER051 |
| Maytenus | guyanensis | oblongata | JQ625799 | JQ626557 |  |
| Melicoccus | pedicellaris | bijugatus | JQ626266 | EU720610 |  |
| Meliosma | veitchiorum | squamulata | AF206793 | DQ401353 |  |
| Meriania | nobilis | NA | AF215533 |  |  |
| Metteniusa | tessmanniana | NA | AM421128 |  |  |
| Miconia | punctata | acuminata | JQ625848 | JQ626538 |  |
| Micrandra | minor | minor | AJ402974 | EF135568 |  |
| Micropholis | guyanensis | guyanensis | JQ626126 | JQ626512 |  |
| Mimosa | spegazzinii | pigra | Z70151 | GU135076 |  |
| Minquartia | guianensis | guianensis | DQ790148 | JQ626517 |  |
| Mirabilis | jalapa | jalapa | M62565 | AY042614 |  |
| Mollinedia | ovata | pinchotiana | AF050218 | GQ429060 |  |
| Monnina | xalapensis | glaberrima | AM234184 | EU604039 |  |
| Morinda | seibertii | officinalis | GQ981805 | GQ434175 |  |
| Moronobea | coccinea | NA | JQ626143 |  |  |
| Morus | alba | alba | L01933 | AB038183 |  |
| Mosannona | vasquezii | vasquezii | AY319064 | AY743508 |  |
| Mouriri | crassifolia | crassifolia | JQ626296 | JQ626576 |  |
| Moutabea | guianensis | guianensis | JQ625841 | JQ626362 |  |
| Mucoa | duckei | NA | KX640869 |  | PER055 |
| Myrcia | fallax | fallax | JQ625851 | AM490006 |  |
| Myrcianthes | fragrans | NA | U26328 |  |  |
| Myrciaria | floribunda | vexator | JQ626319 | AY521544 |  |
| Myriocarpa | longipes | NA | AY208705 |  |  |
| Myrospermum | sousanum | sousanum | U74207 | AY386959 |  |
| Myroxylon | balsamum | balsamum | Z70144 | FJ151488 |  |
| Myrsine | africana | africana | U96652 | AJ429290 |  |
| Naucleopsis | guianensis | NA | JQ626013 |  |  |
| Nealchornea | yapurensis | NA | AY794865 |  |  |
| Nectandra | lineata | lineata | GQ981812 | EU153854 |  |
| Neea | floribunda | floribunda | JQ626040 | FJ037933 |  |
| Norantea | guianensis | guianensis | JQ625952 | JQ626401 |  |
| Ochroma | pyramidale | NA | AJ233118 |  |  |
| Ochthocosmus | longipedicellatus | longipedicellatus | FJ707535 | EF135573 |  |
| Ocotea | bullata | argyrophylla | AM235002 | JQ626566 |  |
| Oenocarpus | bacaba | bacaba | JQ626213 | JQ626537 |  |
| Omphalea | diandra | diandra | AY788183 | FJ670016 |  |
| Onychopetalum | periquino | periquino | AY319065 | AY518876 |  |
| Ophiocaryon | heterophyllum | NA | KX640870 |  | PER059 |
| Opuntia | dillenii | dillenii | AY875233 | AY875369 |  |
| Ormosia | coccinea | coccinea | JQ625915 | GQ982055 |  |
| Osteophloeum | platyspermum | platyspermum | JQ625884 | JQ626371 |  |
| Ouratea | melinonii | NA | JQ625759 |  |  |
| Oxalis | latifolia | latifolia | EU002282 | EU002186 |  |
| Oxandra | asbeckii | espintana | JQ626186 | DQ018260 |  |
| Pachira | dolichocalyx | quinata | JQ626211 | GQ982057 |  |
| Pagamea | coriacea | NA | KX640871 |  | PER064 |
| Palicourea | guianensis | guianensis | JQ625897 | GQ982058 |  |
| Panopsis | cinnamomea | cinnamomea | DQ875850 | EU642708 |  |
| Parahancornia | fasciculata | fasciculata | JQ625735 | DQ660530 |  |
| Parathesis | cubana | NA | AF213814 |  |  |
| Parinari | montana | sp. | JQ625788 | AB233743 |  |
| Parkia | nitida | ulei | JQ626144 | FJ514615 |  |
| Passiflora | quadrangularis | quadrangularis | L01940 | GQ248176 |  |
| Paullinia | venosa | pinnata | AY724365 | AY724320 |  |
| Pausandra | martinii | NA | AY794887 |  |  |
| Pavonia | multiflora | NA | AJ233123 |  |  |
| Paypayrola | grandiflora | grandiflora | AB354429 | AB354501 |  |
| Pedilanthus | tithymaloides | tithymaloides | AY794825 | AB268063 |  |
| Peltogyne | venosa | floribunda | JQ625856 | EU362022 |  |
| Pentagonia | macrophylla | macrophylla | X83658 | GQ982059 |  |
| Pentaplaris | doroteae | NA | AJ233157 |  |  |
| Pera | bicolor | bicolor | AY794968 | EF135578 |  |
| Perebea | guianensis | xanthochyma | JQ626069 | GQ982060 |  |
| Pereskia | aculeata | aculeata | AF206805 | DQ855863 |  |
| Perrottetia | longistylis | longistylis | AY935736 | AY935915 |  |
| Persea | americana | americana | AY337727 | EU153874 |  |
| Petiveria | alliacea | alliacea | AJ402987 | GQ429080 |  |
| Petrea | racemosa | volubilis | U28879 | FJ514600 |  |
| Phyllanthus | acuminatus | acidus | KX640872 | AY936572 | BOLLC358 |
| Phyllostylon | rhamnoides | NA | KX640873 |  | BOLXX091 |
| Physalis | peruviana | peruviana | FJ914180 | EF438898 |  |
| Phytelephas | macrocarpa | macrocarpa | AJ404836 | EF128234 |  |
| Phytolacca | americana | americana | M62567 | DQ401362 |  |
| Picramnia | polyantha | NA | AF127025 |  |  |
| Picrolemma | sprucei | NA | EU043013 |  |  |
| Pilea | depressa | NA | AF500359 |  |  |
| Piper | wallichii | sarmentosum | EF450307 | EF450302 |  |
| Piptadenia | viridiflora | viridiflora | KX640874 | AF521856 | BOLIN205CO |
| Piptocarpha | axillaris | NA | L13651 |  |  |
| Platonia | insignis | NA | JQ626227 |  |  |
| Platymiscium | pinnatum | pinnatum | JQ626063 | JQ626473 |  |
| Platypodium | elegans | elegans | GQ981836 | GQ982065 |  |
| Pleuranthodendron | lindenii | NA | AJ418832 |  |  |
| Plinia | rivularis | NA | JQ626311 |  |  |
| Plumeria | inodora | cubensis | X91767 | DQ660536 |  |
| Podocalyx | loranthoides | loranthoides | AY663647 | EF135583 |  |
| Poecilanthe | effusa | NA | JQ625776 |  |  |
| Pogonophora | schomburgkiana | schomburgkiana | JQ626132 | JQ626518 |  |
| Polygala | hispida | californica | AM234224 | AY386842 |  |
| Poraqueiba | guianensis | guianensis | JQ626039 | JQ626457 |  |
| Porcelia | steinbachii | NA | AY841649 |  |  |
| Posoqueria | latifolia | latifolia | JQ626258 | JQ626556 |  |
| Poulsenia | armata | NA | GQ981838 |  |  |
| Pourouma | bicolor | bicolor | JQ626107 | JQ626509 |  |
| Pouteria | grandis | egregia | JQ625857 | JQ413935 |  |
| Pradosia | cochlearia | ptychandra | JQ626148 | JQ626386 |  |
| Prestoea | acuminata | pubens | AY012487 | AM114648 |  |
| Prestonia | quinquangularis | tomentosa | X91768 | EF456331 |  |
| Protium | altsonii | sagotianum | JQ625813 | JQ626422 |  |
| Prunus | laurocerasus | armeniaca | U06809 | GQ434211 |  |
| Pseudobombax | septenatum | septenatum | GQ981847 | GQ982072 |  |
| Pseudolmedia | laevigata | NA | KX640875 |  | PER071 |
| Pseudomalmea | diclina | diclina | AY841530 | AY841398 |  |
| Pseudopiptadenia | suaveolens | suaveolens | JQ625948 | JQ626397 |  |
| Pseudoxandra | cuspidata | cuspidata | JQ625818 | JQ626358 |  |
| Psidium | friedrichsthalianum | friedrichsthalianum | GQ981848 | GQ982073 |  |
| Psychotria | ficigemma | ficigemma | JQ625868 | JQ626366 |  |
| Pterandra | arborea | arborea | AF344506 | AF344573 |  |
| Pterocarpus | sp. | rohrii | KX640876 | GQ982083 | PER074 |
| Pterogyne | nitens | NA | KX640877 |  | BOLIN100 |
| Ptychopetalum | olacoides | olacoides | JQ626003 | JQ626439 |  |
| Qualea | rosea | rosea | JQ626047 | JQ626462 |  |
| Quararibea | duckei | duckei | JQ626033 | JQ626452 |  |
| Quassia | amara | amara | GQ981863 | AY128207 |  |
| Quercus | serrata | myrsinifolia | AB060576 | AB060063 |  |
| Quiina | obovata | pteridophylla | JQ625761 | EF135589 |  |
| Randia | decemcostata | armata | AJ286700 | GQ982084 |  |
| Rapanea | ferruginea | NA | Z80204 |  |  |
| Rauvolfia | mannii | sellowii | X91769 | DQ660537 |  |
| Recordoxylon | speciosum | NA | JQ626133 |  |  |
| Remijia | pedunculata | pedunculata | AY538506 | AY538417 |  |
| Retiniphyllum | pilosum | NA | AF331654 |  |  |
| Rhabdodendron | amazonicum | amazonicum | JQ625835 | JQ626361 |  |
| Rhamnidium | elaeocarpum | NA | AJ390030 |  |  |
| Rhamnus | cathartica | cathartica | L13189 | AY257533 |  |
| Rhizophora | racemosa | apiculata | AF127690 | AF329466 |  |
| Rhodostemonodaphne | grandis | grandis | JQ626255 | JQ626554 |  |
| Rhynchanthera | grandiflora | NA | AF215542 |  |  |
| Richeria | grandis | grandis | AY663616 | AY830281 |  |
| Rinorea | sp. | sp. | JQ625765 | JQ626350 |  |
| Rivina | humilis | humilis | M62569 | AY042646 |  |
| Rollinia | elliptica | herzogii | JQ626090 | DQ125062 |  |
| Rondeletia | intermixta | NA | AM117264 |  |  |
| Rosenbergiodendron | formosum | formosum | GQ981867 | GQ982087 |  |
| Roucheria | calophylla | NA | FJ169600 |  |  |
| Roupala | macrophylla | montana | AF093728 | EU169661 |  |
| Rourea | minor | minor | FJ707537 | EF135591 |  |
| Roystonea | regia | oleracea | AY012488 | AM114630 |  |
| Ruagea | pubescens | pubescens | DQ238057 | AY128198 |  |
| Rudgea | lorentensis | NA | Z68821 |  |  |
| Ruellia | graezicans | tweediana | L12595 | GU135099 |  |
| Ruizodendron | ovale | NA | AY841657 |  |  |
| Ruizterania | albiflora | albiflora | JQ626202 | JQ626501 |  |
| Ruprechtia | laxiflora | coriacea | EF437987 | AY042648 |  |
| Ruptiliocarpon | caracolito | caracolito | AJ402997 | AY935918 |  |
| Rustia | splendens | NA | Y18716 |  |  |
| Sabal | bermudana | bermudana | AJ404766 | AM114553 |  |
| Sabicea | aspera | aspera | AY538508 | AY538420 |  |
| Sacoglottis | guianensis | cydonioides | JQ626216 | JQ626378 |  |
| Sagotia | racemosa | NA | AY794903 |  |  |
| Salacia | undulata | crassifolia | AJ402998 | FJ705548 |  |
| Salix | magnifica | bebbiana | FJ788570 | EU790690 |  |
| Sambucus | caerulea | racemosa | AJ420867 | AF446898 |  |
| Sanchezia | nobilis | NA | AJ247613 |  |  |
| Sandwithia | guyanensis | NA | JQ626201 |  |  |
| Sapindus | saponaria | saponaria | KX640878 | AY491661 | BOLLC442 |
| Sapium | glandulosum | glandulosum | KX640879 | GQ982089 | PER077 |
| Satyria | warszewiczii | warszewiczii | AF124579 | U61314 |  |
| Saurauia | oldhamii | tristyla | AF088852 | EU310435 |  |
| Sauvagesia | africana | africana | AB233909 | AB233805 |  |
| Scheelea | butyracea | NA | AY044636 |  |  |
| Schefflera | morototoni | decaphylla | JQ625796 | JQ626409 |  |
| Schinopsis | brasiliensis | NA | KX640880 |  | BOLIN047 |
| Schizolobium | parahyba | parahyba | AY904398 | EU362036 |  |
| Schoepfia | schreberi | schreberi | L11205.2 | DQ787447 |  |
| Scleronema | praecox | NA | KX640881 |  | PER078 |
| Sebastiania | pavoniana | NA | AY794840 |  |  |
| Securidaca | bialata | NA | EU644682 |  |  |
| Senna | alata | obtusifolia | U74250 | GQ434282 |  |
| Serjania | communis | communis | AJ403001 | EU720640 |  |
| Sextonia | rubra | rubra | JQ626173 | JQ626456 |  |
| Sida | sp. | NA | AM235027 |  |  |
| Sideroxylon | cinereum | capiri | Z83137 | GQ429074 |  |
| Simaba | cedron | cedron | JQ626166 | JQ626421 |  |
| Simarouba | amara | glauca | JQ626282 | AY128206 |  |
| Simira | wurdackii | NA | KX640882 |  | PER079 |
| Siparuna | decipiens | cristata | JQ626097 | JQ626406 |  |
| Sloanea | guianensis | guianensis | JQ626032 | JQ626451 |  |
| Smilax | glauca | ocreata | AF206822 | GQ434034 |  |
| Socratea | exorrhiza | exorrhiza | KX640883 | AM114618 | PER080 |
| Solanum | tuberosum | lepidotum | M76402 | GQ982099 |  |
| Sorocea | saxicola | affinis | KX640884 | GQ982100 | BOLLC241 |
| Spachea | correae | correae | AF344510 | AF344575 |  |
| Spathelia | excelsa | sp. | AF066798 | FJ716739 |  |
| Spermacoce | hispida | NA | AJ288623 |  |  |
| Spondias | cytherea | mombin | U39274 | AY594480 |  |
| Stachyarrhena | acuminata | acuminata | JQ625826 | JQ626359 |  |
| Stachytarpheta | dichotoma | NA | U32161 |  |  |
| Stemmadenia | littoralis | litoralis | DQ660666 | DQ660542 |  |
| Stenopadus | talaumifolius | talaumifolius | EU385019 | EU385398 |  |
| Sterculia | pruriens | pruriens | JQ625993 | JQ626433 |  |
| Sterigmapetalum | guianense | NA | AF127671 |  |  |
| Stigmaphyllon | puberum | diversifolium | AF344514 | AB233798 |  |
| Struthanthus | woodsonii | woodsonii | EU544474 | EU544458 |  |
| Strychnos | sp. | decussata | JQ626240 | EU214293 |  |
| Stryphnodendron | moricolor | moricolor | JQ626052 | JQ626465 |  |
| Stylogyne | turbacensis | turbacensis | GQ981886 | GQ982104 |  |
| Styrax | pallidus | pallidus | JQ626303 | JQ626577 |  |
| Swartzia | polyphylla | flaemingii | JQ626163 | AY386941 |  |
| Sweetia | fruticosa | fruticosa | KX640885 | AY386911 | BOLIN073 |
| Swietenia | macrophylla | macrophylla | AY128241 | EF489114 |  |
| Syagrus | sancona | smithii | KX640886 | AM114638 | BOLLC118 |
| Symphonia | globulifera | globulifera | JQ625954 | GQ429061 |  |
| Symplocos | martinicensis | martinicensis | JQ625921 | JQ626382 |  |
| Tabebuia | serratifolia | sp. | JQ626306 | JQ626497 |  |
| Tabernaemontana | divaricata | catharinensis | X91772 | DQ660549 |  |
| Tachigali | melinonii | sp. | JQ626276 | EU362054 |  |
| Talauma | ovata | gitingensis | L12666 | AF548642 |  |
| Talisia | hexaphylla | angustifolia | JQ625755 | EU720705 |  |
| Tapirira | obtusa | obtusa | JQ625925 | JQ626383 |  |
| Tapura | guianensis | NA | JQ625895 |  |  |
| Taralea | oppositifolia | NA | KX640887 |  | PER085 |
| Terminalia | sp. | complanata | JQ626332 | GQ248208 |  |
| Ternstroemia | dentata | impressa | JQ625847 | AY336338 |  |
| Tetracera | asiatica | asiatica | AJ235796 | AY042665 |  |
| Tetragastris | panamensis | altissima | JQ626012 | JQ626484 |  |
| Tetrameranthus | laomax | NA | KX640888 |  | PER086 |
| Tetrapterys | microphylla | microphylla | AF344515 | AF344579 |  |
| Tetrastylidium | peruvianum | peruvianum | DQ790154 | DQ790190 |  |
| Tetrathylacium | johansenii | johansenii | GQ981897 | GQ982110 |  |
| Tetrazygia | urbanii | NA | AF215538 |  |  |
| Tetrorchidium | gabonense | rubrivenium | AY794872 | AB268056 |  |
| Theobroma | subincanum | subincanum | JQ626171 | JQ626525 |  |
| Thevetia | peruviana | ahouai | X91773 | GQ982112 |  |
| Thyrsodium | guianense | puberulum | JQ626176 | JQ626480 |  |
| Tibouchina | urvilleana | NA | U26339 |  |  |
| Tococa | rotundifolia | NA | AF215539 |  |  |
| Tocoyena | pittieri | pittieri | GQ981900 | GQ982113 |  |
| Tournefortia | laurifolia | laurifolia | EU599824 | EU599648 |  |
| Touroulia | guianensis | guianensis | JQ625920 | FJ670037 |  |
| Tovomita | calophylla | NA | KX640889 |  | PER087 |
| Trattinnickia | demerarae | aspera | JQ626083 | GQ982114 |  |
| Trema | micrantha | micrantha | KX640890 | GQ982115 | BOLLC352 |
| Trichanthera | gigantea | gigantea | GQ981903 | GQ982116 |  |
| Trichilia | pallida | pallida | JQ626046 | JQ626491 |  |
| Trigonia | nivea | boliviana | AF089761 | AB233744 |  |
| Trigynaea | duckei | lanceipetala | AY841660 | AY743487 |  |
| Triolena | obliqua | NA | AF215515 |  |  |
| Triplaris | poeppigiana | cumingiana | AF297137 | GQ982118 |  |
| Trophis | racemosa | racemosa | KX640891 | GQ982120 | PER088 |
| Trymatococcus | amazonicus | amazonicus | JQ626260 | JQ626558 |  |
| Turnera | ulmifolia | ulmifolia | Z75691 | EF135599 |  |
| Turpinia | occidentalis | occidentalis | KX640892 | GQ982121 | BOLLC394 |
| Unonopsis | rufescens | rufescens | JQ626017 | JQ626445 |  |
| Urera | caracassana | baccifera | KX640893 | GQ982123 | BOLLC355 |
| Vaccinium | macrocarpon | cereum | L12625 | AF419705 |  |
| Vantanea | parviflora | parviflora | JQ625882 | JQ626370 |  |
| Vatairea | erythrocarpa | macrocarpa | JQ625866 | AY386927 |  |
| Vataireopsis | surinamensis | NA | JQ626110 |  |  |
| Verbena | officinalis | officinalis | Z37473 | GQ434146 |  |
| Viburnum | acerifolium | acerifolium | AF446927 | EU749458 |  |
| Viola | decumbens | NA | AM235165 |  |  |
| Virola | kwatae | kwatae | JQ626043 | JQ626460 |  |
| Vismia | cayennensis | billbergiana | JQ626022 | GQ982127 |  |
| Vitex | agnus.castus | trifolia | U78716 | AB284175 |  |
| Vochysia | guianensis | guianensis | JQ625791 | JQ626355 |  |
| Vouacapoua | americana | americana | AY904389 | FJ037922 |  |
| Vouarana | guianensis | NA | JQ626103 |  |  |
| Warszewiczia | cordata | NA | Y18722 |  |  |
| Weinmannia | racemosa | fraxinea | AF307933 | AM889750 |  |
| Wettinia | augusta | hirsuta | KX640894 | AM114619 | PER091 |
| Xylopia | nitida | nitida | JQ626284 | JQ626567 |  |
| Xylosma | benthamii | benthamii | JQ625911 | JQ626379 |  |
| Zanthoxylum | monophyllum | americanum | U39282 | EF489101 |  |
| Zeyheria | tuberculosa | NA | KX640895 |  | BOLIN034 |
| Zinowiewia | australis | concinna | AY935741 | AY935922 |  |
| Ziziphus | ornata | obtusifolia | AJ390052 | AY935939 |  |
| Zygia | racemosa | racemosa | JQ625977 | JQ626423 |  |
