# Supplementary material for: Evolutionary patterns of range size, abundance and species richness in Amazonian angiosperm trees
Source: PeerJ. 2016 Sep 6;4:e2402. doi: 10.7717/peerj.2402 (PMC5018673; doi:10.7717/peerj.2402)
Supplement: Table S2 — The hard minimum age allowed for the node (i.e., the offset) and the mean and standard deviation of the log-normal prior are given, in millions of years. [file peerj-04-2402-s002.docx]

**Table S2:** Nodes in the phylogeny that were constrained in the temporally-calibrated phylogenetic analysis based on fossil ages. The hard minimum age allowed for the node (i.e. the offset) and the mean and standard deviation of a log-normal prior are given in millions of years.

| **Node** | **Minimum age (myrs)** | **Mean age (myrs)** | **Standard deviation (myrs)** |
| --- | --- | --- | --- |
| Apocynaceae stem | 29.8 | 37.5 | 1.9 |
| Brassicaceae stem | 71.4 | 94.1 | 4.5 |
| Caryophyllales crown | 66.8 | 87 | 4.2 |
| Core eudicots stem | 100 | 145.7 | 12.6 |
| Cunoniaceae stem | 66.8 | 87 | 4.2 |
| Ericales crown | 73 | 95.6 | 4.6 |
| Fabales crown | 48 | 61.5 | 3 |
| Fagales stem | 76.8 | 101 | 4.8 |
| Lamiales crown | 35.4 | 44.9 | 2.2 |
| Laurales crown | 84.6 | 112.1 | 5.2 |
| Magnoliales stem | 89.6 | 119.3 | 5.6 |
| Malpighiales crown | 71.2 | 93.2 | 4.5 |
| Malvales crown | 55.8 | 71.9 | 3.5 |
| Myrtales crown | 70.4 | 92 | 4.4 |
| Santalales crown | 41.6 | 52.9 | 2.6 |
| Sapindales crown | 52 | 66.8 | 3.2 |
| Solanales crown | 35.4 | 44.9 | 2.2 |
| Ulmaceae crown | 27.2 | 34.2 | 1.7 |
| Anacardiaceae stem | 52.8 | 67.9 | 3.3 |
| Annonaceae stem | 56 | 72.2 | 3.5 |
| Arecaceae crown | 52 | 66.8 | 3.2 |
| Chrysobalanus stem | 26.4 | 33.2 | 1.7 |
| Combretaceae stem | 59.2 | 76.6 | 3.7 |
| Moraceae stem | 59.2 | 76.6 | 3.7 |
| Sapotaceae stem | 52.8 | 67.9 | 3.3 |
