# Supplementary material for: Evolutionary patterns of range size, abundance and species richness in Amazonian angiosperm trees
Source: PeerJ. 2016 Sep 6;4:e2402. doi: 10.7717/peerj.2402 (PMC5018673; doi:10.7717/peerj.2402)
Supplement: Table S3 — The probability that a given lineage has a smaller value than that reconstructed is also given, based on a permutation test described in the main text. If this probability is smaller than 0.025, then the lineage is described as having a significantly smaller value than expected by chance, while the converse holds if the value is greater than 0.975. [file peerj-04-2402-s003.docx]

**Table S3:** Taxonomic lineages present in the phylogeny along with their reconstructed values for species richness, range size and abundance (all transformed with the natural logarithm prior to analysis). The probability that a given lineage has a smaller value than that reconstructed is also given based on a permutation test described in the main text. If this probability is smaller than 0.025, then the lineage is described as having a significantly smaller value than expected by chance, while the converse holds if the value is greater than 0.975.

| **Lineage** | **Category** | **Reconstructed value**  **log(species richness)** | **Probability of lower value for species richness** | **Reconstructed value**  **log(range size, km^2^)** | **Probability of lower value for range size** | **Reconstructed value**  **log(abundance, # inds)** | **Probability of lower value for abundance** |
| --- | --- | --- | --- | --- | --- | --- | --- |
| Acacieae | tribe | 1.177 | 0.770 | 6.040 | 0.984 | 7.389 | 0.815 |
| Acalyphoideae | subfamily | 0.797 | 0.033 | 5.874 | 0.857 | 7.262 | 0.652 |
| Acanthaceae | family | 1.211 | 0.881 | 5.580 | 0.007 | 6.747 | 0.023 |
| Acanthoideae | subfamily | 1.329 | 0.966 | 5.570 | 0.009 | NA | NA |
| Adoxaceae | family | 1.118 | 0.605 | 5.533 | 0.062 | NA | NA |
| Alchorneae | tribe | 0.637 | 0.068 | 6.156 | 0.991 | 7.657 | 0.935 |
| Alstonieae | tribe | 1.143 | 0.623 | 5.954 | 0.805 | 7.477 | 0.758 |
| Anacardiaceae | family | 0.940 | 0.308 | 5.938 | 0.936 | 7.472 | 0.900 |
| Annonaceae | family | 1.037 | 0.558 | 5.743 | 0.281 | 7.060 | 0.178 |
| Antidesmeae | tribe | 0.971 | 0.464 | 5.805 | 0.531 | 7.334 | 0.647 |
| Apocynaceae | family | 1.029 | 0.500 | 5.859 | 0.782 | 7.095 | 0.240 |
| Apocynoideae | subfamily | 1.278 | 0.841 | 5.680 | 0.199 | NA | NA |
| Aptandraceae | family | 0.541 | 0.021 | 5.801 | 0.513 | 7.493 | 0.850 |
| Aquifoliales | order | 1.186 | 0.826 | 5.651 | 0.051 | 7.111 | 0.332 |
| Arecaceae | family | 0.853 | 0.158 | 5.706 | 0.154 | 7.617 | 0.975 |
| Arecoideae | subfamily | 0.859 | 0.119 | 5.638 | 0.009 | 7.344 | 0.778 |
| Asteraceae | family | 1.426 | 0.912 | 5.434 | 0.007 | NA | NA |
| Asterales | order | 1.489 | 0.994 | 5.413 | 0.001 | NA | NA |
| Asterids | major clade | 1.091 | 0.850 | 5.691 | 0.000 | 7.056 | 0.018 |
| Attaleinae | subtribe | 1.157 | 0.698 | 5.807 | 0.533 | 7.422 | 0.742 |
| Bactridinae | subtribe | 1.237 | 0.811 | 5.643 | 0.116 | 7.101 | 0.358 |
| Beslerieae | tribe | 1.512 | 0.967 | 5.399 | 0.002 | NA | NA |
| Bignoniaceae | family | 1.077 | 0.606 | 5.890 | 0.824 | 7.003 | 0.202 |
| Bixaceae | family | 0.842 | 0.228 | 6.131 | 0.987 | 7.305 | 0.621 |
| Bombacoideae | subfamily | 0.975 | 0.379 | 5.705 | 0.147 | 7.390 | 0.812 |
| Boraginaceae | family | 1.259 | 0.818 | 5.661 | 0.156 | NA | NA |
| Brassicales | order | 1.097 | 0.648 | 5.813 | 0.590 | 7.284 | 0.623 |
| Brongniartieae | tribe | 0.538 | 0.070 | 6.080 | 0.926 | 7.286 | 0.572 |
| Burseraceae | family | 0.855 | 0.168 | 5.934 | 0.895 | 7.336 | 0.676 |
| Byttnerioideae | subfamily | 1.081 | 0.583 | 5.895 | 0.803 | 7.234 | 0.525 |
| Cactaceae | family | 1.080 | 0.553 | 5.462 | 0.035 | NA | NA |
| Calophyllaceae | family | 0.567 | 0.044 | 6.046 | 0.947 | 7.081 | 0.328 |
| Campanulaceae | family | 1.875 | 0.990 | 5.194 | 0.004 | NA | NA |
| Caricaceae | family | 0.993 | 0.479 | 5.827 | 0.578 | NA | NA |
| Caryocaraceae | family | 0.962 | 0.446 | 6.066 | 0.881 | 7.138 | 0.438 |
| Caryophyllales | order | 1.104 | 0.745 | 5.743 | 0.219 | 7.020 | 0.122 |
| Cassieae1 | tribe | 0.961 | 0.378 | 5.941 | 0.884 | 7.031 | 0.222 |
| Cassieae2 | tribe | 0.191 | 0.001 | 6.398 | 0.998 | 8.224 | 0.995 |
| Celastraceae | family | 0.982 | 0.436 | 5.640 | 0.115 | 6.989 | 0.202 |
| Celastrales | order | 0.813 | 0.125 | 5.809 | 0.552 | 7.285 | 0.635 |
| Chiococceae | tribe | 0.582 | 0.053 | 5.947 | 0.831 | 6.892 | 0.198 |
| Chrysobalanaceae | family | 0.962 | 0.421 | 5.920 | 0.844 | 7.396 | 0.751 |
| Cinchoneae | tribe | 1.101 | 0.584 | 5.520 | 0.027 | 6.624 | 0.046 |
| Cinchonoideae | subfamily | 0.843 | 0.075 | 5.633 | 0.004 | 6.535 | 0.000 |
| Clusiaceae | family | 0.830 | 0.122 | 5.837 | 0.647 | 7.377 | 0.807 |
| Clusieae | tribe | 1.249 | 0.809 | 5.625 | 0.104 | 7.189 | 0.453 |
| Cocoseae | tribe | 0.998 | 0.454 | 5.662 | 0.040 | 7.289 | 0.677 |
| Combretaceae | family | 1.362 | 0.855 | 5.951 | 0.798 | 7.174 | 0.463 |
| Condamineeae | tribe | 0.944 | 0.328 | 5.835 | 0.682 | 7.148 | 0.378 |
| Connaraceae | family | 1.380 | 0.781 | 5.883 | 0.685 | 6.559 | 0.081 |
| Coryphoideae | subfamily | 0.472 | 0.016 | 5.812 | 0.564 | 7.079 | 0.347 |
| Crescentieae | tribe | 0.283 | 0.010 | 5.941 | 0.769 | NA | NA |
| Crotonoideae1 | subfamily | 0.755 | 0.002 | 5.975 | 0.992 | 7.503 | 0.975 |
| Crotonoideae2 | subfamily | 0.852 | 0.134 | 5.932 | 0.934 | 7.430 | 0.882 |
| Cucurbitaceae | family | 1.522 | 0.893 | 5.654 | 0.267 | NA | NA |
| Dalbergieae1 | tribe | 1.099 | 0.670 | 5.887 | 0.817 | 7.238 | 0.550 |
| Dalbergieae2 | tribe | 0.944 | 0.353 | 5.792 | 0.465 | 7.347 | 0.725 |
| Detarieae | tribe | 1.132 | 0.694 | 5.733 | 0.299 | 7.388 | 0.787 |
| Dichapetalaceae | family | 1.075 | 0.542 | 5.814 | 0.575 | 7.038 | 0.327 |
| Dipterygeae | tribe | 0.909 | 0.344 | 5.854 | 0.655 | 7.345 | 0.665 |
| Ebenaceae | family | 1.127 | 0.628 | 5.635 | 0.141 | 6.803 | 0.093 |
| Ericaceae | family | 1.162 | 0.644 | 5.405 | 0.022 | NA | NA |
| Ericales | order | 1.131 | 0.818 | 5.597 | 0.000 | 6.942 | 0.027 |
| Erythropalaceae | family | 0.966 | 0.447 | 5.803 | 0.541 | 7.349 | 0.607 |
| Euphorbiaceae | family | 0.764 | 0.002 | 5.989 | 0.997 | 7.469 | 0.981 |
| Euphorbieae | tribe | 0.892 | 0.298 | 5.795 | 0.523 | NA | NA |
| Euphorbioideae | subfamily | 0.731 | 0.010 | 5.953 | 0.969 | 7.626 | 0.990 |
| Euterpeae | tribe | 1.004 | 0.481 | 5.664 | 0.147 | 7.700 | 0.931 |
| Fabaceae | family | 1.077 | 0.671 | 5.863 | 0.876 | 7.384 | 0.931 |
| Fabales | order | 1.099 | 0.774 | 5.843 | 0.813 | NA | NA |
| Gardenieae | tribe | 0.998 | 0.423 | 5.895 | 0.837 | 6.781 | 0.018 |
| Gentianales | order | 1.021 | 0.509 | 5.800 | 0.486 | 6.902 | 0.001 |
| Gesneriaceae | family | 1.441 | 0.968 | 5.482 | 0.006 | NA | NA |
| Grewioideae | subfamily | 0.881 | 0.246 | 5.987 | 0.950 | 7.486 | 0.872 |
| Guettardeae | tribe | 1.106 | 0.664 | 5.596 | 0.020 | 6.613 | 0.084 |
| Hillieae | tribe | 0.751 | 0.161 | 5.699 | 0.250 | NA | NA |
| Hippomaneae | tribe | 0.812 | 0.118 | 5.866 | 0.720 | 7.600 | 0.931 |
| Huerteales | order | 0.553 | 0.076 | 5.821 | 0.571 | NA | NA |
| Humiriaceae | family | 0.799 | 0.223 | 5.839 | 0.650 | 7.360 | 0.689 |
| Ingeae | tribe | 1.219 | 0.866 | 5.846 | 0.685 | 6.983 | 0.133 |
| Iriarteeae | tribe | 0.754 | 0.172 | 5.548 | 0.039 | 7.848 | 0.979 |
| Ixonanthaceae | family | NA | NA | NA | NA | 7.572 | 0.790 |
| Ixoroideae | subfamily | 1.061 | 0.599 | 5.809 | 0.582 | 6.667 | 0.000 |
| Jatropheae | tribe | 0.798 | 0.234 | 5.852 | 0.653 | NA | NA |
| Lacistemataceae | family | NA | NA | NA | NA | 6.467 | 0.048 |
| Lamiaceae | family | 1.062 | 0.606 | 5.776 | 0.408 | 6.698 | 0.037 |
| Lamiales | order | 1.066 | 0.628 | 5.635 | 0.005 | 6.790 | 0.008 |
| Lauraceae | family | 0.947 | 0.363 | 5.713 | 0.239 | 6.929 | 0.118 |
| Laurales | order | 1.242 | 0.902 | 5.582 | 0.010 | 6.962 | 0.095 |
| Lecythidaceae | family | 0.634 | 0.027 | 5.943 | 0.871 | 7.893 | 0.997 |
| Linaceae | family | 0.674 | 0.131 | 6.153 | 0.971 | 7.388 | 0.710 |
| Loganiaceae | family | 0.985 | 0.454 | 5.953 | 0.848 | 6.896 | 0.105 |
| Lythraceae | family | 1.332 | 0.846 | 5.965 | 0.856 | NA | NA |
| Magnoliaceae | family | 0.908 | 0.415 | 5.360 | 0.020 | NA | NA |
| Magnoliales | order | 1.172 | 0.921 | 5.657 | 0.009 | 7.185 | 0.431 |
| Magnoliids | major clade | 1.348 | 0.998 | 5.614 | 0.003 | 7.030 | 0.099 |
| Malpighiaceae | family | 1.189 | 0.786 | 5.738 | 0.317 | 6.799 | 0.080 |
| Malpighiales | order | 0.937 | 0.079 | 5.876 | 0.983 | 7.316 | 0.918 |
| Malvaceae | family | 1.023 | 0.480 | 5.895 | 0.909 | 7.362 | 0.817 |
| Malvales | order | 0.943 | 0.267 | 5.979 | 0.983 | 7.313 | 0.714 |
| Malvoideae | subfamily | 1.186 | 0.829 | 5.724 | 0.217 | NA | NA |
| Manihoteae | tribe | 1.219 | 0.737 | 5.872 | 0.656 | NA | NA |
| Mauritiinae | subtribe | 0.608 | 0.141 | 5.807 | 0.543 | 8.504 | 0.994 |
| Melastomataceae | family | 1.461 | 0.998 | 5.718 | 0.173 | 6.920 | 0.060 |
| Meliaceae | family | 0.938 | 0.300 | 5.829 | 0.630 | 7.537 | 0.944 |
| Menispermaceae | family | 0.829 | 0.296 | 6.029 | 0.862 | 6.323 | 0.023 |
| Millettieae | tribe | 1.227 | 0.699 | 5.689 | 0.316 | NA | NA |
| Mimoseae | tribe | 1.256 | 0.895 | 5.960 | 0.936 | 7.619 | 0.913 |
| Mimosoideae | subfamily | 1.183 | 0.868 | 5.960 | 0.974 | 7.342 | 0.797 |
| Monocots | major clade | 1.205 | 0.844 | 5.673 | 0.088 | NA | NA |
| Moraceae | family | 0.789 | 0.053 | 5.943 | 0.952 | 7.601 | 0.985 |
| Myristicaceae | family | 1.180 | 0.716 | 5.803 | 0.525 | 7.897 | 0.980 |
| Myrtaceae | family | 1.517 | 0.990 | 5.844 | 0.652 | 6.643 | 0.013 |
| Myrtales | order | 1.300 | 0.996 | 5.827 | 0.696 | 6.995 | 0.033 |
| Nyctaginaceae | family | 0.983 | 0.445 | 5.637 | 0.120 | 6.983 | 0.268 |
| Ochnaceae | family | 0.975 | 0.374 | 5.897 | 0.844 | 7.195 | 0.481 |
| Olacaceae | family | 0.523 | 0.030 | 5.827 | 0.608 | 7.262 | 0.541 |
| Oxalidales | order | 1.393 | 0.991 | 5.642 | 0.023 | 7.097 | 0.281 |
| Papilionoideae | subfamily | 0.852 | 0.036 | 5.854 | 0.848 | 7.384 | 0.928 |
| Passifloraceae | family | 1.646 | 0.991 | 5.788 | 0.483 | NA | NA |
| Phaseoleae | tribe | 1.228 | 0.833 | 5.786 | 0.474 | NA | NA |
| Phyllanthaceae | family | 0.881 | 0.179 | 5.935 | 0.943 | 7.305 | 0.737 |
| Phyllantheae | tribe | 0.917 | 0.390 | 6.140 | 0.957 | 7.371 | 0.675 |
| Phytolaccaceae | family | 0.680 | 0.055 | 5.910 | 0.829 | NA | NA |
| Piperales | order | 1.630 | 0.998 | 5.579 | 0.015 | NA | NA |
| Plumerieae | tribe | 0.910 | 0.292 | 5.863 | 0.726 | 6.938 | 0.275 |
| Polygalaceae | family | 1.312 | 0.881 | 5.831 | 0.588 | NA | NA |
| Polygonaceae | family | 1.372 | 0.867 | 5.766 | 0.444 | 7.013 | 0.295 |
| Primulaceae | family | 1.094 | 0.670 | 5.437 | 0.000 | 6.602 | 0.002 |
| Prockieae | tribe | 0.242 | 0.005 | 6.076 | 0.898 | 7.945 | 0.956 |
| Proteaceae | family | 1.150 | 0.657 | 5.369 | 0.002 | 7.434 | 0.741 |
| Psychotrieae | tribe | 1.864 | 0.999 | 5.694 | 0.222 | 6.548 | 0.009 |
| Rauvolfioideae1 | subfamily | 0.984 | 0.361 | 5.817 | 0.583 | 7.091 | 0.242 |
| Rhamnaceae | family | 0.924 | 0.303 | 5.797 | 0.488 | 7.188 | 0.470 |
| Rhamneae | tribe | 0.738 | 0.209 | 5.755 | 0.401 | NA | NA |
| Rhizophoraceae | family | 0.859 | 0.245 | 5.709 | 0.237 | 6.843 | 0.093 |
| Rosales | order | 1.017 | 0.484 | 5.773 | 0.307 | 7.270 | 0.704 |
| Rosids | major clade | 1.049 | 0.679 | 5.772 | 0.162 | 7.201 | 0.435 |
| Rubiaceae | family | 0.997 | 0.392 | 5.833 | 0.756 | 6.868 | 0.001 |
| Rubioideae | subfamily | 1.283 | 0.981 | 5.777 | 0.402 | 6.718 | 0.005 |
| Ruellieae | tribe | 1.438 | 0.974 | 5.606 | 0.056 | NA | NA |
| Ruelliinae | subtribe | 1.310 | 0.843 | 5.664 | 0.202 | NA | NA |
| Rutaceae | family | 0.943 | 0.334 | 5.626 | 0.047 | 6.939 | 0.128 |
| Sabiaceae | family | 1.129 | 0.623 | 5.558 | 0.117 | 7.040 | 0.336 |
| Salicaceae | family | 0.616 | 0.001 | 6.087 | 0.998 | 7.468 | 0.933 |
| Samydeae | tribe | 0.560 | 0.005 | 6.151 | 0.998 | 7.596 | 0.953 |
| Santalales | order | 0.642 | 0.003 | 5.846 | 0.727 | 7.347 | 0.776 |
| Sapindaceae | family | 1.026 | 0.518 | 5.945 | 0.908 | 7.067 | 0.279 |
| Sapindales | order | 0.944 | 0.156 | 5.869 | 0.936 | 7.294 | 0.781 |
| Sapotaceae | family | 0.968 | 0.387 | 5.535 | 0.014 | 7.216 | 0.520 |
| Simaroubaceae | family | 0.591 | 0.060 | 5.944 | 0.849 | 7.352 | 0.667 |
| Solanaceae | family | 1.348 | 0.945 | 5.719 | 0.250 | 6.537 | 0.011 |
| Solanales | order | 1.400 | 0.977 | 5.728 | 0.247 | NA | NA |
| Solanoideae | subfamily | 1.643 | 0.997 | 5.689 | 0.218 | NA | NA |
| Spermacoceae | tribe | 1.185 | 0.672 | 5.870 | 0.674 | NA | NA |
| Swartzieae | tribe | 0.807 | 0.144 | 5.786 | 0.448 | 7.382 | 0.809 |
| Symphonieae | tribe | 0.385 | 0.007 | 6.116 | 0.971 | 7.750 | 0.957 |
| Tabermontantaneae | tribe | 0.870 | 0.227 | 5.828 | 0.616 | 7.125 | 0.378 |
| Teucrioideae | subfamily | 1.353 | 0.808 | 5.623 | 0.188 | NA | NA |
| Ulmaceae | family | 0.521 | 0.078 | 6.049 | 0.886 | 7.860 | 0.933 |
| Urticaceae | family | 1.143 | 0.765 | 5.730 | 0.245 | 7.057 | 0.267 |
| Vataireoid | tribe | 0.491 | 0.023 | 6.056 | 0.937 | 7.422 | 0.700 |
| Verbenaceae | family | 1.202 | 0.892 | 5.724 | 0.195 | NA | NA |
| Violaceae | family | 0.980 | 0.389 | 5.941 | 0.910 | 7.561 | 0.928 |
| Vochysiaceae | family | 1.295 | 0.848 | 5.772 | 0.436 | 7.177 | 0.449 |
| Willughbeeae | tribe | 0.883 | 0.316 | 5.807 | 0.529 | 7.489 | 0.786 |
